# Natural mating ability is associated with gut microbiota composition and function in captive male giant pandas

**DOI:** 10.1002/ece3.11189

**Published:** 2024-04-01

**Authors:** Zheng Yan, Yinghu Lei, Pengpeng Zhao, Danhui Zhang, Jiena Shen, Guiquan Zhang, Rongping Wei, Haoqiu Liu, Xiaoyan Liu, Yan He, Sijia Shen, Dingzhen Liu

**Affiliations:** ^1^ Department of Ecology, College of Life Sciences, Key Laboratory for Biodiversity and Ecological Engineering of Ministry of Education Beijing Normal University Beijing Hebei China; ^2^ Research Center for the Qinling Giant Panda Shaanxi Rare Wildlife Rescue Base Xi'an Shaanxi China; ^3^ China Conservation and Research Centre for the Giant Panda Wolong Sichuan China

**Keywords:** behavior, breeding, genetic diversity, giant panda, gut microbiota, hormone

## Abstract

The issue of poor sexual performance of some male giant pandas seriously impairs the growth and the genetic diversity of the captive population, yet there is still no clear understanding of the cause of the loss of this ability and its underlying mechanism. In this study, we analyzed the gut microbiota and its function in 72 fecal samples obtained from 20 captive male giant pandas, with an equal allocation between individuals capable and incapable of natural mating. Additionally, we investigated fecal hormone levels and behavioral differences between the two groups. A correlation analysis was then conducted among these factors to explore the influencing factors of their natural mating ability. The results showed significant differences in the composition of gut microbiota between the two groups of male pandas. The capable group had significantly higher abundance of *Clostridium* sensu stricto *1* (*p*_adjusted_ = .0021, GLMM), which was positively correlated with fatty acid degradation and two‐component system functions (Spearman, *p*_adjusted_ < .05). Additionally, the capable group showed higher gene abundance in gut microbiota function including purine and pyrimidine metabolism and galactose metabolism, as well as pathways related to biological processes such as ribosome and homologous recombination (DEseq2, *p*_adjusted_ < .05). We found no significant differences in fecal cortisol and testosterone levels between the two groups, and no difference was found in their behavior either. Our study provides a theoretical and practical basis for further studying the behavioral degradation mechanisms of giant pandas and other endangered mammal species.

## Graphical abstract

This study investigated the gut microbiota of captive male giant pandas and found significant differences between the microbiota of individuals’ capable and incapable of natural mating. The findings provide insight into potential mechanisms affecting the reproductive success of endangered mammal species.

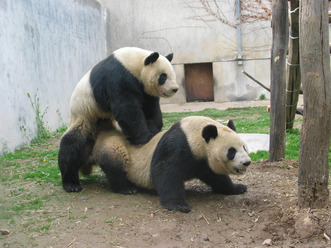

## 1 INTRODUCTION

Mating and reproduction are instinctive behaviors, yet for some captive animals, especially males in the order Carnivora, sexual performance is poor and natural reproductive rates are low. For example, cheetahs (*Acinonyx jubatus*) (Marker & O'Brien, 1989), polar bears (*Ursus maritimus*) (Clubb & Mason, 2007), clouded leopards (*Neofelis nebulosa*) (Tipkantha et al., 2017), and giant pandas (*Ailuropoda melanoleuc*a) (Peng et al., 2009; Zhang et al., 2021) display poor sexual behavior under captive conditions. The underlying causes, however, remain largely unknown. Research on the poor sexual behavior of captive male animals can be summarized into the following aspects. First, the rearing history hypothesis proposes that the various non‐natural stressors in the captive environment, such as confined spaces and noise, all contribute to high stress levels and may cause stereotypic behaviors in animals, which in turn may lead to poor sexual behavior and low sexual motivation in those animals (Hodel et al., 2021; Martin et al., 2020; Peng et al., 2007; Zhang et al., 2004). Second, the free competition hypothesis states that in the wild, males often compete for mating opportunities with females. However, in captivity, the absence of competition may lead to the loss of mate choice. This may result in psychological distress and a subsequent lack of interest in mating (Nie et al., 2015; Peng et al., 2009; Swaisgood & Schulte, 2010). Third, the nutrition hypothesis suggests that the weak mating ability in captive males may be attributed to inadequate nutrition. In captivity, animals lack the freedom to choose optimal food, potentially resulting in insufficient intake of trace elements essential for reproduction. As a consequence, this may adversely affect digestion, absorption, and the production of metabolic byproducts, which may in turn lead to a negative impact on their reproductive performance (Zhang, Wang, Ayala, et al., 2022; Zhang, Wang, Zhang, & Hou, 2022). Recently, there is a growing scholarly interest in exploring the potential correlation between gut microbiota and male‐mating ability.

Microbes can influence metabolism, immunity, and behavior (O'Mahony et al., 2017). Specific changes in hormone levels are also associated with the presence of gut microbiota (Neuman et al., 2015). Due to the observed association between microbial characteristics and testosterone levels (Karakas & Surampudi, 2018), the gut microbiota is also considered a potential biomarker for male hypogonadism. The brain and gut microbiota maintain a constant bidirectional connection through various neural, endocrine, immune, and metabolic pathways, known as the microbiota gut brain axis (MGBA) (O'Mahony et al., 2017). Previous research has found that the gut microbiota influences the levels of host pheromones, which in turn affect mating behavior. For example, *Lactobacillus plantarum* determines the mating preference of *Drosophila melanogaster* and leads them to mate with individuals that have the same gut microbiota composition (Sharon et al., 2010, 2011). In recent years, the microbiota gut testis axis (MGTA) has been proposed, in which the gut microbiota regulates the immune microenvironment of the testes by providing nutrients such as short‐chain fatty acids, vitamins, and minerals (Li et al., 2022; Li, Qi, et al., 2017). Through signal transduction, it controls physiological processes involved in changing the function and gene expression status of the reproductive system by microbial metabolism or de novo synthesis of key molecules (Dai et al., 2015; Yan et al., 2023; Zhang, Sun, Geng, et al., 2022). These molecules have nutritional, immune, and hormone‐related functions, and promote the male reproductive system through the circulatory system (Cai et al., 2022). In conclusion, the health status or balance of the gut microbiota also affects the development and health of the mammalian male reproductive system (Martinot et al., 2022). This influence can be either positive or negative in nature (Guo et al., 2020; Liu et al., 2021).

The giant panda is a vulnerable species of ursid endemic to China (Swaisgood et al., 2003). The wild population is distributed among six fragmented mountainous areas in Sichuan, Shaanxi, and Gansu provinces (Wei et al., 2015). Captive breeding has played an important role in the giant panda ex situ conservation (Swaisgood et al., 2017). The first successful natural breeding of captive giant pandas occurred in 1963 at Beijing Zoo. However, only 12 males and 21 females were predominately breeding in captivity from 1936 to 1998 (Peng et al., 2001). The problem of poor natural mating ability in captive male pandas still persists (Zhang et al., 2021). Alternative methods such as artificial insemination have been used to propagate the species; however, excessive reliance on this technique has a lower birth rate compared to natural mating (<30%) (Li, Wintle, et al., 2017). Furthermore, female pandas that have undergone artificial insemination may exhibit reduced maternal care and an increased likelihood of rejecting cubs (Swaisgood et al., 2022). Therefore, the poor mating ability of male giant pandas impairs the genetic diversity of the captive population.

The giant panda possesses unique foraging characteristics as a member of the order Carnivora that feeds primarily on bamboo, which is low in nutrition but high in fiber content (Wang et al., 2017). The giant panda lacks the gene for digesting cellulose, the main component of bamboo (Li et al., 2010), and relies on the key function of gut microbiota to aid in bamboo digestion (Zhu et al., 2011). However, to our knowledge, there is currently no literature available on the effects of gut microbiota on sexual behavior and mating ability in this species. Therefore, the main objective of this study was to test the hypothesis that gut microbiota, cortisol, and testosterone levels, as well as daily behavior, affect the natural mating ability of male giant pandas. Specifically, we conducted a study on 20 captive adult male giant pandas, with an equal ratio (1:1) of individuals being capable and incapable of natural mating, focusing on the gut microbiota, fecal hormones, and animal behavior. We analyzed the correlations among these factors. The results will not only provide a theoretical and practical basis for further studying the mechanisms of behavioral degeneration in giant pandas and other endangered mammalian species but also contribute to improving the genetic quality and diversity of captive populations as a whole. This, in turn, may help identify high‐quality candidates for future reintroduction programs and support the construction of the Giant Panda National Park and biodiversity conservation efforts.

## 2 MATERIALS AND METHODS

### 2.1 Subjects and samples collection

**FIGURE 1:**
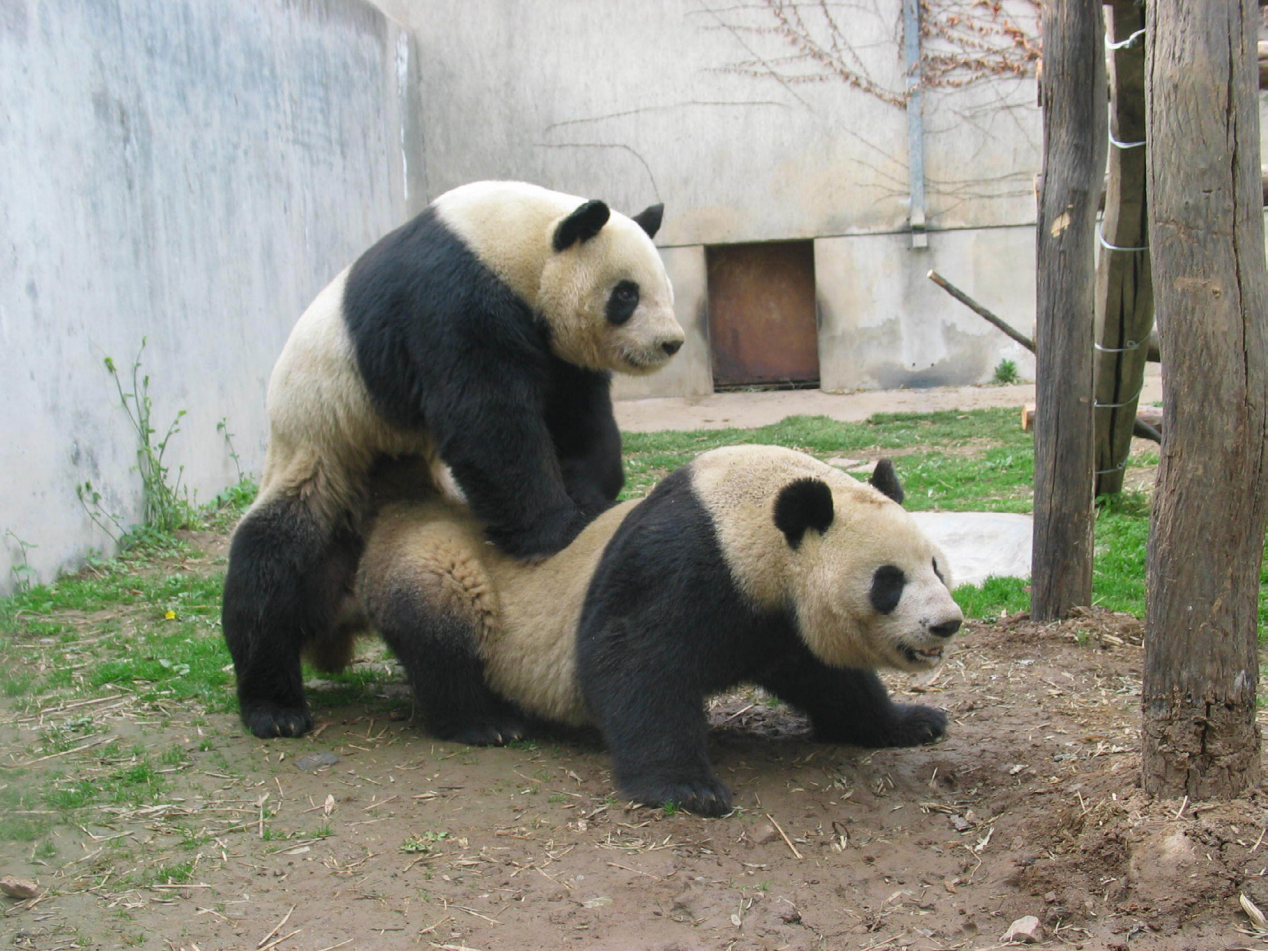
Captive giant pandas engaging in natural mating at RCQGP.

We collected fecal samples from captive adult male giant pandas. Those capable of autonomously mating with estrous female giant pandas, completing the insertion and ejaculation, were classified into the capable of natural mating group (capable group, Figure 1); otherwise, they were classified into the incapable of natural mating group (incapable group). All pandas were individually housed, with each enclosure consisting of an indoor resting area and an outdoor activity space. The outdoor area featured artificial wooden structures for enrichment, as well as small ponds for free access to water. The subjects had unrestricted access to fresh bamboo and were supplemented with small amounts of apples and a corn‐based supplement. For detailed information about the husbandry and management, please see the description of previous researches (Liu et al., 2003; Wang et al., 2017; Zhang, Dong, Zhao, et al., 2022). During the experimental period, no antibiotics were administered to the giant pandas. All protocols for animal management were approved by the Animal Welfare and Ethics Committee of Beijing Normal University.

In March 2023, twenty‐one fecal samples were collected from seven pandas (with a ratio of capable to incapable as 4:3) in the Research Center for the Qinling Giant Panda (RCQGP), Shaanxi Rare Wildlife Rescue Base. Additionally, fifteen fecal samples were collected from four pandas (with a ratio of capable to incapable as 3:1) at Shenshuping panda base of the China Conservation and Research Center for the Giant Panda (CCRCGP) at Wenchuan, Sichuan, China. Fecal samples were collected within 10 min after defecation, where the outer layer of feces in contact with the ground was removed. The samples were then placed in sterile bags, air was expelled, and we used a cooler box to store them in a −80°C freezer within 20 min. To increase the sample size, sequencing data (NCBI BioProject PRJNA433781) from 36 fecal samples of 10 pandas (with a ratio of capable to incapable as 3:7) from previously published dataset located in the Chengdu Research Base of Giant Panda Breeding (CRBGPB) were also included in our analysis. The sample collection, preservation, and sequencing methods for this dataset were consistent with the procedures described in this study, ensuring consistency in subsequent data analysis. Ultimately, a total of 72 fecal samples from 20 giant pandas were included in the analysis for this study. Among them, the capable group comprised 10 individuals, producing 28 fecal samples, with an average age of 15.1 years (SD: 3.11, range: 9–21). The incapable group comprised 10 individuals (one of which was relocated from CRBGPB to RCQGP and was sampled in both locations), producing 44 fecal samples, with an average age of 12.5 years (SD: 3.68, range: 8–18). All individuals were of normal mating age at the time of sampling (Martin‐Wintle et al., 2019).

### 2.2 DNA extraction and 16S rRNA gene‐sequencing processing

We used a DNA extraction kit (Tiangen, Beijing, China) to perform DNA extraction from fecal samples. The V4 region of the 16S rRNA gene was amplified with 515F and 806R primers. We used the NEB Next® Ultra™ II FS DNA PCR‐free Library Prep Kit from New England Biolabs (Ipswich, MA, USA) for library construction. We used Qubit and Q‐PCR to quantify the constructed libraries. After passing quality control, we sequenced them on the Illumina NovaSeq PE 250 platform (Illumina, San Diego, California, USA), provided by Novogene Biological Information Technology Co (Beijing, CN), which generated 250 bp paired‐end reads.

Based on the barcode sequences and PCR amplification primers, the raw sequencing data were demultiplexed to separate the data of each sample. We used the DADA2 pipeline to analyze the sequences obtained from gut microbiotas (Callahan et al., 2016) (v.1.28.0) in R (v.4.3.1). This pipeline, based on the divisive amplicon denoising algorithm, was employed to infer amplicon sequence variants (ASVs). The initial step involved filtering and trimming the reads. Quality filtering was performed, and reads containing N bases were removed. A maximum of two errors per forward and reverse read was allowed. Subsequently, the DADA2 algorithm used a parametric error model (err) to estimate error rates for both forward and reverse reads. The estimated error rates were visually inspected and deemed reasonable, ensuring confidence in subsequent steps (Appendix S1). Dereplication was then performed on the forward and reverse reads to eliminate redundancy. The core sample inference algorithm was applied to further quality control, utilizing the error model. To obtain the full denoised sequences, the forward and reverse reads were merged. All 72 sequences that were generated have been uploaded to the sequence read archive under the BioProject ID PRJNA1003259.

Subsequently, an amplicon ASV table was constructed, and chimeras were removed. We used the SILVA rRNA gene reference database (v.138) for taxonomic classification of the ASVs (Quast et al., 2013). ASVs assigned to Eukarya, Chloroplasts, or Mitochondria, as well as those of unknown Kingdom origin, were excluded from the dataset. As a result, the dataset consisted of 5332 remaining ASVs. On average, samples retained over 76% (SE 1.9%) of their total sequences after processing in DADA2. Appendix S2 showed the statistical outcomes achieved at each stage of the data processing pipeline.

### 2.3 Gut microbiota diversity and composition analysis

Unless otherwise specified, statistical analysis and data visualization were performed in R software. The bioinformatics analysis followed the methods of previous research with slight modification (Wen et al., 2023), and the main steps are listed below. Prior to alpha‐diversity analyses, we used the R *phyloseq* (v1.44.0) package to control potential sequencing depth influences by subsampling all samples to 17,098 sequences (McMurdie & Holmes, 2013). We used the R *microbiome* (v.1.22.0) package to construct rarefaction curves of ASV richness and sequencing depth (Lahti & Shetty, 2023). The curves reached a plateau, indicating that the majority of species in the community were observed and suitable for characterizing fecal microbiota profiles. We used the R *vegan* package (v.2.6.4) to calculate microbial alpha‐diversity indices (Oksanen et al., 2007). Simpson's evenness was used to measure species evenness in the community. Shannon diversity index was used to measure community diversity. Pielou's evenness index was used to assess the relationship between species richness and evenness in the community, with higher values indicating higher species richness and evenness (Willis, 2019). To calculate and compare beta‐diversity among different communities, Bray–Curtis distance, Weighted UniFrac, and Unweighted UniFrac were used as distance metrics. Principal coordinates analysis (PCoA) was performed to transform multidimensional data into a low‐dimensional space, followed by analysis of similarities (Adonis) to analyze community differences with a significance threshold of .05. Finally, the significance of community differences was determined based on the set significance threshold. We used the R *ggplot2* package (v.3.4.2) to conduct the visualization of the aforementioned diversity indices (Wickham, 2011).

We used the R *ggalluvial* package (v.0.12.5) to calculate microbial abundance at the phylum and genus levels (Brunson, 2020). The *ggplot2* package was used to generate a species composition‐stack bar diagram comparing the capable and incapable groups, displaying the top ten gut microbiota by abundance. To visualize the hierarchical clustering of samples based on Bray–Curtis distance, the *ggtree* package (v.3.8.0) (Xu et al., 2022) was used. Complete linkage clustering was applied for the clustering analysis. The resulting hierarchical clustering circle tree provides a visual representation of the differences between samples.

### 2.4 Prediction of gut microbial gene function

We used the *Tax4Fun2* package (v.1.1.5) for the microbial functional prediction of the giant pandas (Wemheuer et al., 2020). The *Tax4Fun2* package is a tool that enables rapid prediction of functional profiles and redundancy in prokaryotes based on 16S rRNA gene sequences. The program utilizes the basic local alignment search tool (BLAST) (v2.14.0) (Boratyn et al., 2013) to align gene sequences against the Ref100NR reference database. Ref100NR is a non‐redundant protein sequence database derived from the National Center for Biotechnology Information (NCBI) reference sequence database (RefSeq) (O'Leary et al., 2016). It provides a curated subset of annotated protein sequences with reduced redundancy for protein sequence analysis and bioinformatics applications. We used the Kyoto Encyclopedia of Genes and Genomes (KEGG) Orthology database for functional annotation. The KEGG Orthology (KO) database is widely used for molecular functional reference and can be employed for functional annotation of most microorganisms (Kanehisa et al., 2016). Based on the functional characteristics of the species' genomes and the abundance information provided by the ASV abundance table, community functional abundance can be inferred. The minimum similarity threshold for mapping to the reference data is set at 97%.

Normalization was performed by calculating the average 16S rRNA copy number for each sequence in the reference database. The functional genes were then mapped to their respective KO functions (KEGG level 4 classification unit) and the associated KEGG pathway (KEGG level 3 classification unit). We used the *ggpicrust2* package (v1.7.2) for statistical analysis and visualization after obtaining the community functional abundance table (Yang et al., 2023).

### 2.5 Fecal cortisol and testosterone assay

Fecal cortisol and testosterone level assay, as well as behavioral observations and video recordings, were conducted on seven male giant pandas at the RCQGP. Among them, the proportion of individuals being capable and incapable of natural mating was 4:3. We used the same method as described above to collect one fecal sample from each individual for three consecutive days. Thus, resulting in a total of 21 samples. Fecal samples used for hormone assay were the same as those used for microbial analysis, ensuring enhanced credibility for the correlation analysis between fecal hormones and microbes. We used a radioimmunoassay (RIA) diagnostic kit (Beijing North Biotechnology Company, Beijing, China) following the method of previous research to examine and analyze the fecal cortisol and testosterone levels (Deng et al., 2014). We used a vacuum freeze dryer (LGJ‐10N, Yaxingyi, Xian, China) to freeze‐dry the fecal samples at −60°C for 72 h. Then the samples were ground and sieved. Next, a 5 mL of 80% methanol solution was used to shake and extract the hormones from the samples.

We used the iodine [^125^I] cortisol radioimmunoassay (RIA) kit (sensitivity = 2 pg/mL, coefficient of variation (CV) = 3.24%) to determine the fecal cortisol content, and the [^125^I] testosterone RIA kit (sensitivity = 0.02 ng/mL, CV = 6.69%) to determine the fecal testosterone content. Both kits were obtained from Beijing North Institute of Biological Technology (Beijing, China), and the XH6080 radioimmunoassay analyzer (Xi'an Nuclear Instrument Factory, Xi'an, China) was used for hormone quantification.

### 2.6 Behavioral observation

The behavior of seven captive giant pandas at the RCQGP were observed and recorded while they freely roamed in the outdoor area. Continuous video recordings were conducted from 9:00 am to 4:00 pm each day, which corresponded to the time when the giant pandas were active in the outdoor area. This resulted in a total of 49 h of video data. Except for one giant panda with severely missing data (>30% missing data), the behavioral data of other giant pandas are relatively complete, with an average of 96.4% (SE 1%) valid data. Therefore, the behavioral data of six pandas were used for analysis. The ratio of capable to incapable for natural mating was 1:1. A total of 42 h of behavior data (7 h per day for each individual) were analyzed for further investigation. Following the behavioral definitions of previous research (Liu et al., 2003), a total of 12 behaviors were observed and recorded (Appendix S3). Among them, behaviors such as sniffing, anogenital marking, urine marking, and gazing opposite sex were categorized as reproductive‐related behaviors, while behaviors such as investigating, playing, grooming, and defecation, which were not the focus of attention, were categorized as other behaviors. The focal animal sampling and continuous recording methods were employed throughout the entire observation period (Liu et al., 2006). Ultimately, six behaviors were included for subsequent statistical analysis: i.e., feeding, resting, moving, stereotypic behavior, reproductive behavior, and other behaviors. The duration of feeding, resting, and moving behaviors was recorded in minutes, with 1 min as the unit. If multiple behaviors occurred within a one‐minute unit, the behavior with the highest proportion of time was recorded. All other behaviors, excluding the aforementioned three, were recorded based on frequency (Appendix S3).

### 2.7 Statistics analysis

We used the *ggClusterNet* package (v.0.1.0) (Wen et al., 2022) and the *tidyverse* package (v.2.0.0) (Wickham et al., 2019) to cluster and weight the ASV table at the phylum and genus levels, retaining the top 200 abundant microbial phyla and genera. We used the trimmed mean of M‐values (TMM) method to normalize the data and correct for expression biases caused by differences in sequencing depth and sample variations. In the analysis of microbial diversity differences between capable and incapable groups, as well as variations in microbial composition at the phylum and genus levels and hormone differences, we used the R *lme4* package (v.1.1–35.1) (Bates et al., 2015). Due to repeated measures on individual subjects existed in the dataset, a generalized linear mixed model (GLMM) was used to examine the data. Whether the male panda could naturally mate or not was entered as a fixed factor categorical variable in the GLMMs, and the dependent variables were hormones measures or microbial measures. The subject identity was entered as a random factor in the GLMMs. For visual representation, the *ggplot2* package and the *Patchwork* package (v.1.1.2) (Pedersen, 2022) were used to plot extended error bars’ plot, displaying the top 30 differentially abundant microbes. We used the DESeq2 method to perform differential analysis of the functional genes in the gut microbiota of giant panda feces. The top 30 significantly differentially abundant metabolic pathways were selected, and then we used the *ggprism* package (v.1.0.4) to generate combined bar plot (Dawson, 2022).

To solve the small sample size problem, we used the bootstrap statistical method to augment the sample size of our behavioral data (Dwivedi et al., 2017), with 5000 repetitions. Mean differences for each sample were calculated using the bootstrap test method, and *p*‐values were computed based on these samples to assess the significance of observed differences. We used the *tidyverse* package, rstatix (v0.7.2) (Kassambara, 2023b), *ggpubr* (v0.6.0) (Kassambara, 2023a), and *ggtext* (v0.1.2) (Wilke & Wiernik, 2022) for data analysis and visualization.

The correlation analysis was conducted on the enriched top 10 abundant taxa at the genus level, phylum level, and top 30 differentially expressed KEGG pathways. Since the data did not meet normal distribution according to the Shapiro–Wilk test, Spearman's rank correlation analysis was used. Finally, features with an absolute correlation coefficient >.7, ranked in the top 30 in terms of abundance, and showing significant correlations were selected. We used the Wekemo Bioincloud platform (https://www.bioincloud.tech) to generate a correlation network plot. We used the Benjamini–Hochberg correction to adjust the raw *p*‐values obtained from multiple comparisons with the significance threshold set at the adjusted *p*‐value of .05 in this study.

## 3 RESULTS

### 3.1 Microbial diversity and compositional differences

**FIGURE 2:**
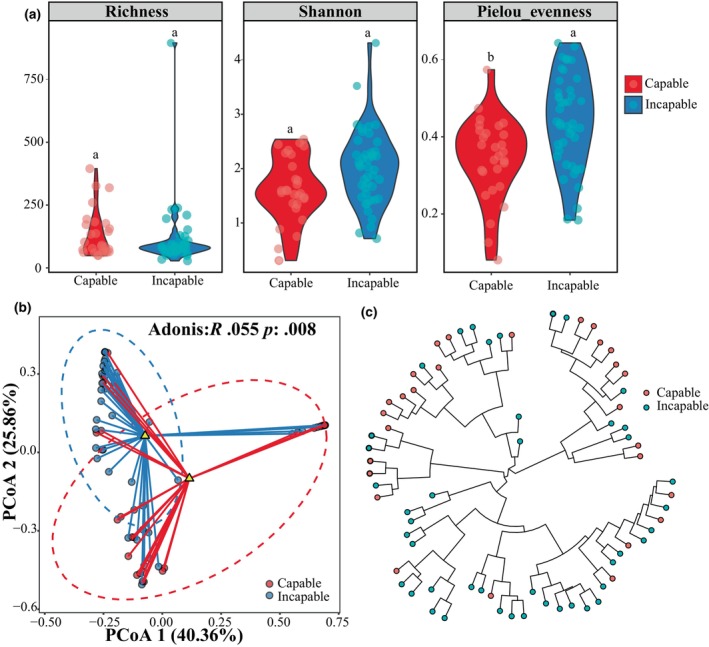
Gut microbiota diversity and hierarchical clustering of captive male giant pandas. (a) Alpha diversity levels represented by Richness, Shannon, and Pielou's evenness diversity indices, with different letters indicating significant differences (*p* < .05). (b) Beta diversity PCoA plot based on Bray–Curtis distance and intergroup Adonis dissimilarity index. *p* < .05 indicates significant differences. (c) Hierarchical clustering circle tree, where each node represents a taxonomic unit, and the branch length indicates the degree of variation within the corresponding species.

In this study, a total of 72 fecal samples from captive giant pandas were subjected to clustering analysis, resulting in the identification of 5332 Amplicon Sequence Variants (ASVs) that were annotated to 56 phyla and 730 genera. We found significant differences in microbial diversity between the capable group and the incapable group of natural mating. The capable group exhibited significantly lower values of Pielou's evenness (*p* = .0132, GLMM) compared to the incapable group, but Richness and Shannon index showed no significant between two groups (Figure 2a). Beta diversity analysis based on Bray–Curtis distance revealed distinct intergroup differences in microbial community composition (*p* = .008, Adonis) (Figure 2b). Additionally, PCoA plots based on Weighted UniFrac and Unweighted UniFrac distances demonstrated significant separation between the two groups *p* = .017 and *p* = .001, respectively, Adonis (Appendix S4). The dendrogram of gut microbiota similarity reflects host phylogeny, and the leaf nodes in the branches indicate distinct separation between the two groups (Figure 2c).

**FIGURE 3:**
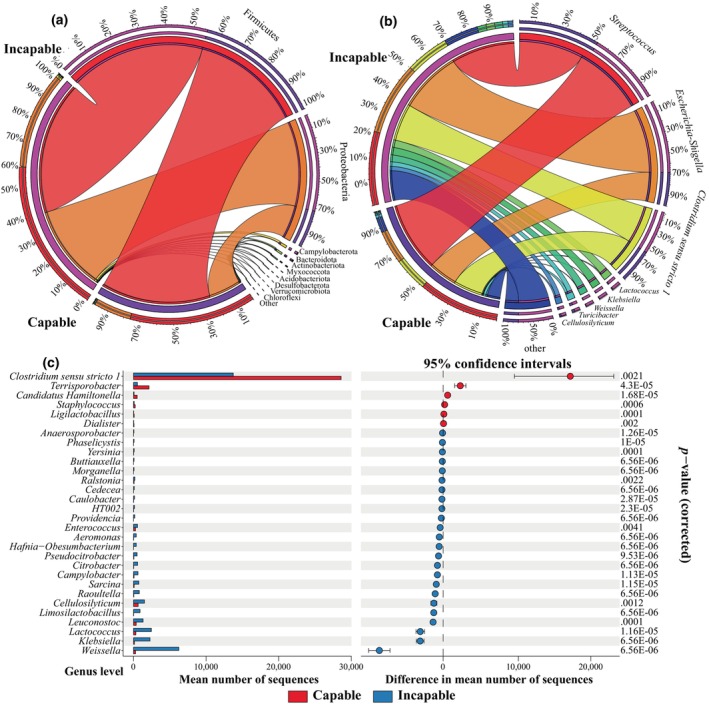
Composition of gut microbiota in captive male giant pandas. Composition of microbial phyla (a) and genera (b) between the capable and incapable groups. (c) We performed a difference analysis at the genus level between the two groups and used an extended error bar plot in the style of Statistical Analysis of Metagenomic Profiles (STAMP) for visualization. The top 30 differentially abundant genera with significant differences are shown. The left side displays a bar plot comparing the average abundance between groups, and the right side shows scatter plots of the average abundance and 95% confidence intervals. The *p*_adjusted_ values were all <.05.

At the phylum level, both groups were predominantly composed of Firmicutes, accounting for over 50% of all bacterial phyla, followed by Proteobacteria (Figure 3a). The capable group exhibited a higher relative abundance of Firmicutes compared to the incapable group, but not reach statistical significance (*p*_adjusted_ > .05, GLMM), while Proteobacteria was significantly lower in the capable group (*p*_adjusted_ = .039, GLMM). The composition of microbial genera between the two groups is shown in Figure 3b. *Streptococcus* and *Escherichia‐shigella* had the highest relative abundances in the capable and incapable groups, respectively. The third‐ranked genus was *Clostridium* sensu stricto *1*, which was significantly higher in the capable group compared to the incapable group (*p*_adjusted_ = .0021, GLMM) (Figure 3c). Additionally, *Terrisporobacter*, *Candidatus Hamiltonella*, *Staphylococcus*, *Ligilactobacillus*, and *Dialister* were significantly higher in the capable group. On the other hand, *Weissella*, *Lactococcus*, *Klebsiella*, *Leuconostoc*, *Cellulosilyticum*, and *Limosilactobacillus* were significantly higher in the incapable group (*p*_adjusted_ < .05, GLMM) (Figure 3c).

### 3.2 The functional differences of gut microbiota

**FIGURE 4:**
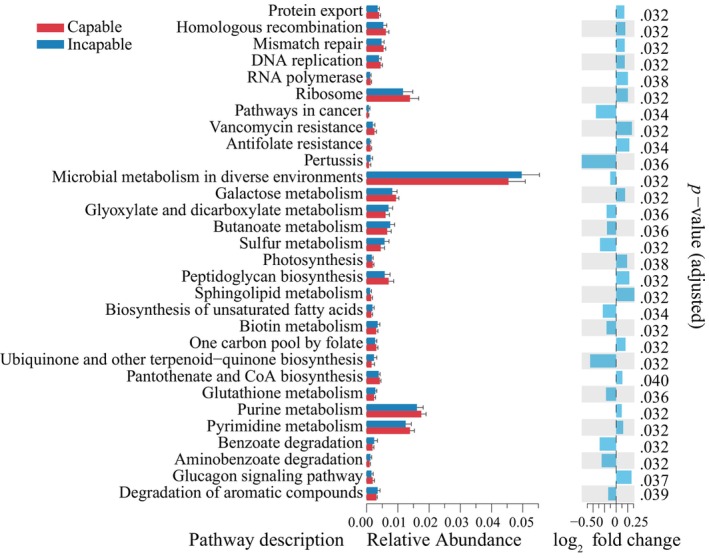
Differential prediction of gut microbiota gene functions between capable and incapable male giant pandas. The bar plot on the left represents the top 30 KEGG pathways with significant differences in relative abundance (*p*_adjusted_ < .05). The fold change plot on the right illustrates the differential changes between the two groups.

In the capable group, pathways such as purine metabolism, pyrimidine metabolism, ribosome, galactose metabolism, peptidoglycan biosynthesis, homologous recombination, mismatch repair, DNA replication, pantothenate and CoA biosynthesis, protein export, and one carbon pool by folate were significantly higher (*p*_adjusted_ < .05, DESeq2) than those in the incapable group. In contrast, the incapable group showed significantly higher abundance (*p*_adjusted_ < .05, DESeq2) in pathways such as microbial metabolism in diverse environments, butanoate metabolism, glyoxylate and dicarboxylate metabolism, sulfur metabolism, biotin metabolism, degradation of aromatic compounds, glutathione metabolism, benzoate degradation, and ubiquinone and other terpenoid‐quinone biosynthesis (Figure 4).

### 3.3 The correlation between gut microbiota and gene annotation functions

**FIGURE 5:**
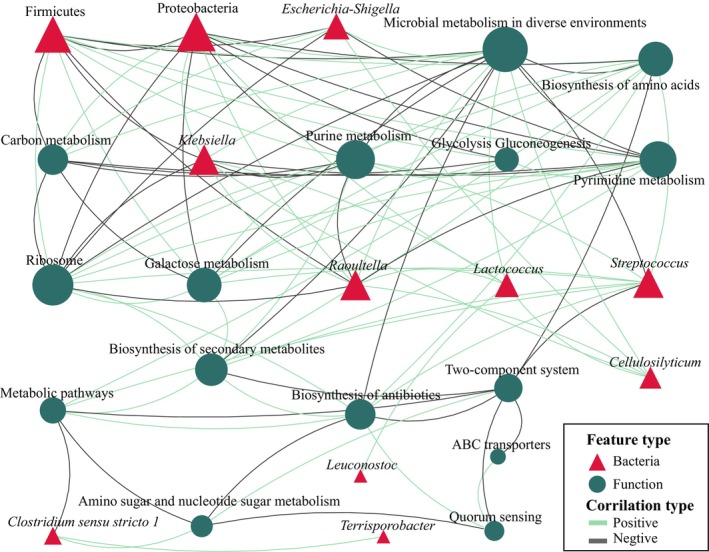
The correlation between the gut microbiota and gene predicted functions in captive adult male giant pandas. For data readability, only the top 30 features with an absolute correlation value >.7 are displayed. All data nodes can be found in Appendix S5. The correlations were calculated using Spearman's rank correlation coefficient, and significance was determined with a *p*_adjusted_ value of <.05. The size of the nodes represents the relative abundance, with larger nodes indicating higher abundance.

The significantly higher abundance of *Clostridium* sensu stricto *1* in the capable group showed significant correlations with four microbial species and 22 metabolic pathways (*p*_adjusted_ < .05, Spearman's rank correlation, same as follow). Among them, *Terrisporobacter* showed the highest correlation (*r* = .71), followed by fatty acid degradation (*r* = .67) and two‐component system (*r* = .65). Similarly, the significantly higher abundance of *Staphylococcus* in the capable group showed significant correlations with 27 microbial species and 31 KEGG pathways. The highest correlations were observed with *Ralstonia* (*r* = .84), *Anaerosporobacter* (*r* = .76), *Cedecea* (*r* = .74), *Lactococcus* (*r* = .73), *Raoultella* (*r* = .72), and *Cellulosilyticum* (*r* = .72). In terms of gene functions, *Staphylococcus* showed the highest correlation with carbon fixation in photosynthetic organisms (*r* = −.73) and pentose phosphate pathway (*r* = −.71). Furthermore, the significantly higher abundance of *Ligilactobacillus* in the capable group showed the highest correlation with caulobacter (*r* = .62) (Figure 5 and Appendix S5).

In the incapable group, the significantly higher abundance of *Weissella* showed significant correlations with 48 features, but the absolute values of these correlations were all below .5. However, both *Klebsiella* and *Lactococcus*, which were significantly higher abundance in the incapable group, exhibited a strong positive correlation (*r* = .82). They also showed a positive correlation with *Raoultella*, *Citrobacter*, *Cedecea*, *Pseudocitrobacter*, and *Anaerosporobacter* with correlationship coefficients >.8. Moreover, the two bacteria showed a positive correlation with correlationship coefficients >.8 with seven functional features, including sulfur metabolism, ubiquinone and other terpenoid‐quinone biosynthesis, microbial metabolism in diverse environments, and pertussis. *Klebsiella* also exhibited a positive correlation >.7 with pathways in cancer and butanoate metabolism. *Lactococcus* showed significant correlations with 59 features, and a positive correlation >.7 with glutathione metabolism (Figure 5 and Appendix S5).

### 3.4 Fecal hormone levels and behavioral differences

**FIGURE 6:**
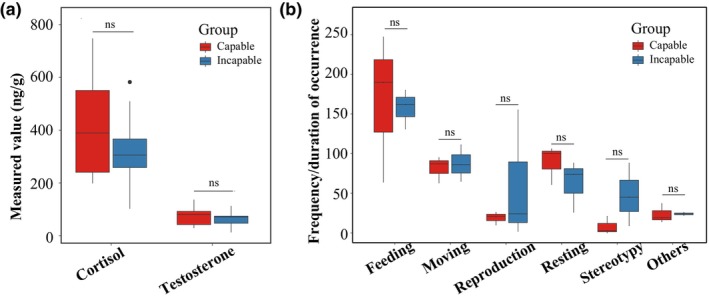
Differences in (a) cortisol and testosterone levels and (b) behaviors between the capable group and incapable group of giant pandas for natural mating. “ns” indicates non‐significant differences.

There were no significant differences in fecal cortisol and testosterone levels between the two groups of giant pandas (Figure 6a) (*p* > .05, GLMM). During the study period, a total of 12 behaviors were recorded for the giant pandas (see Appendix S3 for details). In total, 2482 valid data points were obtained from the six giant pandas. However, there were no significant differences observed in the six main behaviors between these two groups of male pandas (Figure 6b) (*p*_adjusted_ > .05, Bootstrap test).

## 4 DISCUSSION

Although some previous studies have focused on the reproductive issues of captive male giant pandas (Martin‐Wintle et al., 2017; Swaisgood et al., 2000; Zhang, Wang, Ayala, et al., 2022), to our knowledge, this is the first study to comprehensively analyze the factors influencing the natural mating ability of captive adult male giant pandas from the perspectives of gut microbiota and its functions, fecal hormones, and behavior, as well as their interrelationships. Our results confirmed the hypothesis that gut microbiota is one of the factors that affect the natural mating ability of captive male giant pandas. However, we did not find impact on natural mating ability from testosterone and cortisol hormone levels, as well as behavior performances during non‐breeding periods.

The result of alpha diversity of gut microbiota suggest that both capable and incapable groups exhibit similar species richness, but the incapable group exhibited significantly higher Pielou's evenness value, indicating a more even distribution and balance of species within the gut microbiota compared to the capable group (Willis, 2019). Based on the Bray–Curtis distance, weighted unifrac, and unweighted unifrac analyses, significant differences were observed in beta diversity between the two groups, indicating distinct differences in species composition and similarity between samples (Ricotta et al., 2019). Furthermore, the results of the hierarchical clustering tree also demonstrated clear differences in similarity between the two groups of samples. Therefore, there were significant differences in the composition of gut microbiota between captive male giant pandas with and without natural mating ability.

The composition of microbiota at various taxonomic levels revealed that Firmicutes and Proteobacteria were the predominant phyla, while *Streptococcus*, *Escherichia*‐*shigella*, and *Clostridium* sensu stricto *1* were the major genera. This finding is consistent with previous studies (Cui et al., 2023; Jin et al., 2020; Yan et al., 2021). Although Firmicutes were more abundance in the capable group, there was no significant difference observed between the two groups at the phylum level. Within Firmicutes, however, *Clostridium* sensu stricto *1* was significantly more abundant in the capable group. Prior phylogenetic studies, based on sequencing of the 16S rRNA gene, suggested that the genus *Clostridium* should be restricted to *Clostridium* cluster I, also known as *Clostridium* sensu stricto (Cruz‐Morales et al., 2019). Therefore, in previous studies on giant pandas, *Clostridium* sensu stricto *1* is referred as *Clostridium* (Huang et al., 2022; Tang et al., 2020). *Clostridium* is widely present in the gastrointestinal tract of various mammals and has been extensively studied. It can metabolize and produce short‐chain fatty acids (Zhang et al., 2015) and plays a vital role in maintaining intestinal immune homeostasis (Rajilić‐Stojanović & de Vos, 2014). Previous research has shown that *Clostridium ramosum* regulates enterochromaffin cell development and serotonin release (Mandić et al., 2019), while *Clostridium scindens* converts glucocorticoids to androgens, a group of male steroid hormones, through side‐chain cleavage (Ridlon et al., 2013). Furthermore, the latest study classified wild, reintroduction, and captive giant pandas into three community clusters of gut microbiota (enterotypes), specifically *Escherichia*, *Clostridium*, and *Pseudomonas* enterotypes (Huang et al., 2023). Our results indicated that the incapable group had a higher abundance of *Escherichia*, while the capable group exhibited an enrichment of *Clostridium*. Thus, the capable group displayed an enterotype transition towards a wild‐like enterotype. However, further studies are required to investigate the role of *Clostridium* in the natural mating ability of captive male giant pandas. Another significant taxon within Firmicutes, *Terrisporobacter*, was also more abundant in the capable group. Similar to *Clostridium* sensu stricto *1*, *Terrisporobacter* is involved in fiber degradation, glucose metabolism, and production of short‐chain fatty acids (Bauchart‐Thevret et al., 2009). As a lactate‐utilizing bacterium, *Terrisporobacter* ferments glucose to produce acetate and CO_2_ under anaerobic conditions (Deng et al., 2015). Additionally, *Ligilactobacillus*, significantly higher in the capable group, is a lactic acid bacterium that has been developed as a probiotic for the treatment and prevention of gastrointestinal diseases in animals, serving as an alternative to antibiotics (Kumar et al., 2022). These microbial taxa in the giant panda's gastrointestinal tract may play a role in maintaining gut homeostasis, aiding nutrient metabolism, and enhancing male reproductive ability.

To explore the impact of gut microbiota changes, we used gene function annotation methods to predict the functional pathways of these gut microbiota and identify the significantly higher relative abundance of functional pathways in the capable group. Among them, purine and pyrimidine metabolism is crucial for providing necessary energy and cofactors to promote cell survival and proliferation, playing a vital role in controlling various aspects of cellular biochemical reactions (Kundu & Dubey, 2021; Pedley & Benkovic, 2017). Additionally, metabolic pathways such as ribosome, homologous recombination, mismatch repair, and DNA replication play important roles in biological processes, contributing to cell growth, development, metabolism, and genetics (Gavande et al., 2016; Pelletier et al., 2018). Furthermore, we found that fatty acid degradation and two‐component system in the metabolic pathways were significantly positively correlated with *Clostridium* sensu stricto *1*. Fatty acid degradation plays a major role in energy production in various cell types (Teng et al., 2016), and the two‐component system is a signaling mechanism in bacteria and other microorganisms that plays a crucial role in microbial adaptation and survival. It is involved in regulating bacterial growth, metabolism, tolerance, pathogenicity, and other aspects (Nguyen & Hong, 2008). Therefore, *Clostridium* sensu stricto *1* plays an important role in maintaining gut homeostasis, metabolic capacity, and signal transduction in the gastrointestinal tract of captive adult male giant pandas. Research has revealed that in captive conditions, prolonged consumption of bamboo shoots can increase the relative abundance of *Clostridium* sensu stricto *1* in the gut microbiota of giant panda (Yan et al., 2021). The ingestion of flavonoid compounds can also achieve this effect (Wang et al., 2021). Furthermore, bamboo shoots have a higher protein content compared to culms and leaves (Wang et al., 2017). The intake of protein has been found to positively affect male‐mating behavior, mating success rate, and sperm quality (Droney, 1996; Schumacher et al., 2014). Additionally, dietary flavonoid compounds are important natural prebiotics for maintaining the dynamic balance of the gut microbiota (Ivey et al., 2019; Wang et al., 2021; Yan et al., 2021).

Testosterone, as an important male hormone, is directly related to male sexual performance (Galansky et al., 2022). The results of this study showed no significant difference in testosterone levels between the two groups of giant pandas. This may be due to the fact that the samples were collected during non‐breeding periods. A previous study in wild pandas at Qinling Foping Nature Reserve has shown that testosterone levels peak during the mating season to meet the demands of courtship and fertilization, while during other periods, testosterone levels remain a comparatively low level because maintaining high levels of testosterone metabolism requires more energy (Nie et al., 2012). Further research during the mating season is needed to provide a more comprehensive understanding of the role of testosterone in maintaining mating ability. In addition, the fecal cortisol levels also showed no significant difference between the two groups. This could be attributed to the sampling location in the Research Center for the Qinling Giant Panda, where the giant pandas reside at a higher altitude with outdoor enclosures situated in the mountains. The average temperature during sampling was around 15°C, which is considered to be suitable for giant pandas (Liu et al., 2016). Additionally, the park receives fewer visitors, reducing potential sources of stress (Deng et al., 2014). As a result, the overall stress levels were relatively low, leading to the non‐significant differences between the two groups.

Contrary to our expectation, we found no significant behavioral differences in the six main behaviors between the two groups of pandas. Regarding reproductive behavior, we speculate that the absence of signals from female pandas, such as urine and anogenital gland secretions, during non‐mating season (Swaisgood et al., 2000; Zhang et al., 2004) led to minimal display of mating behaviors by the male pandas in our study. This may have obscured the mating behaviors of sexually active males, resulting in the lack of differences observed. As for moving behavior, we believe that the limited duration of our observations (less than a week) may not have been sufficient to reflect noticeable difference. To better understand the physical activity of capable naturally mating giant pandas, longer term behavioral data would be necessary. Regarding resting behavior, based on our experience, some captive pandas spend several minutes to hours feeding during the night, so a more comprehensive data collection on resting time would be needed.

Our research provides guidance for further investigation into the behavioral degradation mechanisms of giant pandas and conservation efforts for other endangered mammal species. A comprehensive understanding of the relationship between gut microbiota and animal behavior, and physiology will provide management strategies and conservation measures for protecting endangered species and promote the development of this field.

## 5 CONCLUSION

This study primarily investigated the factors influencing the natural mating ability of captive giant panda and found that the gut microbiota plays a significant role. Specifically, *Clostridium* sensu stricto *1* was identified as having a positive impact on male pandas' natural mating ability, *Clostridium* sensu stricto *1* is associated with fatty acid degradation and plays important roles in maintaining gut homeostasis, metabolic capacity, and signal transduction. However, this study found no evidence in the daily levels of cortisol and testosterone hormones reflecting those male pandas' mating ability, nor were their daily behaviors. By referring to previous studies (Ivey et al., 2019; Schumacher et al., 2014; Wang et al., 2017; Yan et al., 2021, 2024) and current results, we recommend the following measures to improve the captive male pandas' natural ability. (1) To increase and prolong the feeding amount and time of bamboo shoots as wild ones do each year. (2) To add dietary flavonoid compounds in their daily routine diet especially before and during the mating season.

## AUTHOR CONTRIBUTIONS

**Zheng Yan:** Conceptualization (lead); data curation (lead); formal analysis (lead); investigation (lead); software (lead); validation (lead); visualization (lead); writing – original draft (lead); writing – review and editing (lead). **Yinghu Lei:** Conceptualization (equal); formal analysis (equal); resources (equal); supervision (equal). **Pengpeng Zhao:** Conceptualization (equal); data curation (equal); formal analysis (equal); resources (equal). **Danhui Zhang:** Investigation (equal); resources (equal). **Jiena Shen:** Resources (equal); supervision (equal). **Guiquan Zhang:** Resources (equal); supervision (equal). **Rongping Wei:** Resources (equal); supervision (equal). **Haoqiu Liu:** Investigation (equal); resources (equal). **Xiaoyan Liu:** Data curation (equal); investigation (equal). **Yan He:** Writing – review and editing (equal). **Sijia Shen:** Writing – review and editing (equal). **Dingzhen Liu:** Data curation (equal); funding acquisition (lead); project administration (lead); resources (equal); supervision (lead); writing – review and editing (equal).

## FUNDING INFORMATION

This work was supported by National Natural Science Foundation of China (Grant#32270506, 31772466, 31472009), and National Forestry and Grassland Administration, P. R. China (2019‐BJ018).

## CONFLICT OF INTEREST STATEMENT

The author declares that there are no competing financial interests or personal biases that could affect the content of this article.

## Supporting information


Appendix S1‐S5.

## Data Availability

All sequences that were generated have been uploaded to the Sequence Read Archive under the Bio Project ID PRJNA1003259.
