# Supplementary material for: Natural mating ability is associated with gut microbiota composition and function in captive male giant pandas
Source: Ecol Evol. 2024 Apr 1;14(4):e11189. doi: 10.1002/ece3.11189 (PMC10985376; doi:10.1002/ece3.11189)
Supplement: Supplementary file 1 — Appendix S1‐S5. [file ECE3-14-e11189-s001.docx]

**Natural mating ability is associated with Gut Microbiota Composition and Function in Captive Male Giant Pandas**

**Supplementary information**


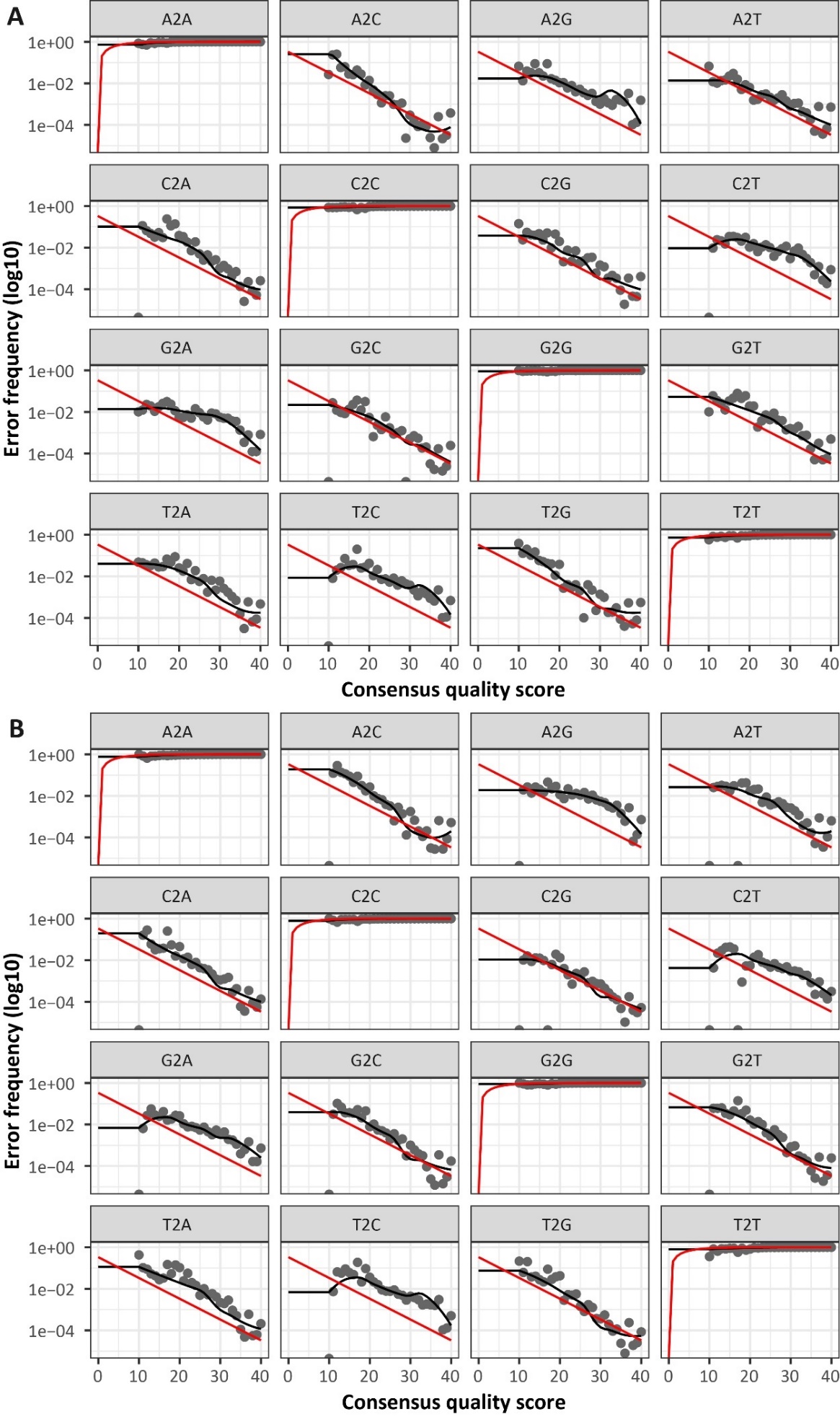


Appendix S1 The error rates for forward (A) and reverse (B) reads as visualized by the DADA2 algorithm using the parameter error model (err). The error rates for each possible transition (A→C, A→G, etc.) are depicted. The points represent the observed error rates for each consensus quality score. The black line represents the estimated error rates after convergence of the machine-learning algorithm. The red line represents the error rates expected under the nominal definition of the Q-score. In this case, the estimated error rates (black line) closely match the observed rates (points), and the error rates decrease with increased quality as expected.

Appendix S2 Statistical results obtained at each step of the data processing process

| Sample | input | filtered | denoisedF | denoisedR | merged | nonchim | excluded |
| --- | --- | --- | --- | --- | --- | --- | --- |
| Male1 | 92378 | 84711 | 84183 | 84208 | 83619 | 82733 | 17273 |
| Male2 | 97316 | 88268 | 87663 | 87634 | 87092 | 86207 | 43028 |
| Male3 | 71903 | 67895 | 66591 | 66899 | 65239 | 63816 | 61482 |
| Male4 | 98855 | 95664 | 95379 | 95408 | 93309 | 91910 | 86960 |
| Male5 | 88351 | 85945 | 85633 | 85700 | 83731 | 82125 | 81578 |
| Male6 | 95238 | 92519 | 92294 | 92347 | 90714 | 90238 | 89893 |
| Male7 | 99980 | 96973 | 96685 | 96661 | 94685 | 92383 | 88987 |
| Male8 | 67764 | 62846 | 62489 | 62464 | 62066 | 61157 | 40927 |
| Male9 | 60676 | 56669 | 55757 | 55879 | 54788 | 54234 | 36126 |
| Male10 | 84261 | 77496 | 76800 | 76865 | 75737 | 74630 | 46444 |
| Male11 | 140174 | 117936 | 117302 | 117316 | 116077 | 114182 | 109350 |
| Male12 | 132393 | 105910 | 105617 | 105657 | 105289 | 104876 | 101559 |
| Male13 | 135814 | 112210 | 111839 | 111814 | 111196 | 109843 | 107353 |
| Male14 | 70698 | 68689 | 68350 | 68320 | 66317 | 65249 | 65098 |
| Male15 | 94416 | 91582 | 91230 | 90973 | 88728 | 87513 | 85982 |
| Male16 | 90887 | 88181 | 87869 | 87942 | 86076 | 85291 | 78951 |
| Male17 | 88605 | 86188 | 85953 | 85978 | 84021 | 82581 | 78561 |
| Male18 | 99591 | 96484 | 96181 | 96242 | 94607 | 93291 | 79551 |
| Male19 | 93545 | 90644 | 90341 | 90368 | 88762 | 87449 | 83245 |
| Male20 | 96239 | 93371 | 93077 | 93084 | 91061 | 89188 | 78260 |
| Male21 | 83236 | 80756 | 80430 | 80487 | 78711 | 77360 | 64025 |
| Male22 | 80182 | 77845 | 77571 | 77450 | 75692 | 74683 | 71247 |
| Male23 | 85473 | 82951 | 82490 | 82616 | 80336 | 78974 | 61172 |
| Male24 | 68979 | 66814 | 66527 | 66461 | 64694 | 63987 | 62523 |
| Male25 | 87046 | 84326 | 84090 | 84020 | 82479 | 81632 | 78659 |
| Male26 | 102070 | 91533 | 90090 | 90318 | 88583 | 86772 | 83082 |
| Male27 | 74064 | 67863 | 67113 | 67022 | 66114 | 64970 | 49920 |
| Male28 | 82232 | 72728 | 71963 | 71984 | 70891 | 69639 | 66472 |
| Male29 | 102137 | 93634 | 89083 | 88871 | 84910 | 83660 | 49100 |
| Male30 | 84471 | 79078 | 78292 | 78329 | 76862 | 75515 | 42780 |
| Male31 | 71098 | 65939 | 65651 | 65573 | 65133 | 64533 | 40757 |
| Male32 | 107884 | 95778 | 94543 | 94646 | 92762 | 91143 | 61904 |
| Male33 | 82394 | 73240 | 72747 | 72730 | 71967 | 70800 | 56070 |
| Male34 | 55441 | 51774 | 51548 | 51571 | 51073 | 50605 | 27530 |
| Male35 | 66959 | 60747 | 60238 | 60207 | 59560 | 59214 | 38278 |
| Male36 | 102600 | 94023 | 91782 | 91968 | 89622 | 87810 | 65985 |
| Male37 | 76464 | 70677 | 69787 | 69920 | 69048 | 68044 | 32283 |
| Male38 | 97729 | 94903 | 94295 | 94151 | 90824 | 86964 | 85751 |
| Male39 | 83170 | 80614 | 80295 | 80219 | 78330 | 71783 | 69692 |
| Male40 | 83586 | 81042 | 80684 | 80708 | 78321 | 69683 | 67175 |
| Male41 | 89222 | 86592 | 86305 | 86274 | 84309 | 81903 | 80903 |
| Male42 | 95305 | 92707 | 92355 | 92301 | 89691 | 86651 | 79763 |
| Male43 | 61870 | 60023 | 59774 | 59717 | 58312 | 57228 | 56422 |
| Male44 | 81479 | 79187 | 78938 | 78918 | 77149 | 75117 | 74746 |
| Male45 | 84951 | 82583 | 82001 | 82092 | 78026 | 71072 | 69952 |
| Male46 | 94010 | 91547 | 91180 | 91196 | 88531 | 86166 | 85858 |
| Male47 | 99745 | 96496 | 96102 | 96003 | 93618 | 92192 | 79080 |
| Male48 | 70222 | 68087 | 67791 | 67744 | 66512 | 64924 | 60432 |
| Male49 | 86197 | 83732 | 83447 | 83471 | 81597 | 80531 | 78362 |
| Male50 | 92599 | 89558 | 88842 | 89021 | 85174 | 84184 | 80338 |
| Male51 | 85489 | 82931 | 82550 | 82696 | 81271 | 80591 | 77843 |
| Male52 | 64068 | 61185 | 60154 | 59966 | 58569 | 56334 | 55405 |
| Male53 | 96246 | 92932 | 92571 | 92641 | 91624 | 90442 | 41847 |
| Male54 | 89759 | 87020 | 86619 | 86546 | 84373 | 81349 | 78944 |
| Male55 | 82107 | 79719 | 79429 | 79408 | 77224 | 74692 | 73691 |
| Male56 | 84231 | 82152 | 81772 | 81737 | 79500 | 77787 | 77561 |
| Male57 | 90277 | 87699 | 87346 | 87385 | 84969 | 80606 | 79479 |
| Male58 | 134531 | 111184 | 110556 | 110559 | 109490 | 106934 | 105215 |
| Male59 | 144775 | 121309 | 121027 | 120984 | 120658 | 118696 | 92579 |
| Male60 | 136814 | 104297 | 104074 | 104045 | 103662 | 102143 | 102054 |
| Male61 | 135905 | 110553 | 110373 | 110374 | 110140 | 108177 | 107073 |
| Male62 | 136027 | 111776 | 111362 | 111505 | 110892 | 107572 | 105743 |
| Male63 | 134992 | 111124 | 110940 | 110967 | 110634 | 106200 | 105607 |
| Male64 | 115943 | 98499 | 98003 | 98104 | 97050 | 94305 | 91785 |
| Male65 | 132383 | 107381 | 107125 | 107170 | 106661 | 105362 | 84259 |
| Male66 | 133141 | 110492 | 110070 | 110112 | 109192 | 106674 | 103104 |
| Male67 | 136211 | 117145 | 116616 | 116718 | 115568 | 113907 | 102730 |
| Male68 | 112640 | 95352 | 95037 | 95080 | 94531 | 93118 | 91425 |
| Male69 | 79288 | 67811 | 67530 | 67594 | 67153 | 66482 | 65470 |
| Male70 | 83017 | 71765 | 71092 | 71086 | 70012 | 69319 | 60265 |
| Male71 | 67595 | 63287 | 62490 | 62410 | 61406 | 60454 | 36491 |
| Male72 | 73275 | 68815 | 68498 | 68517 | 68248 | 67515 | 17098 |

Note: input: The number of original sequences. filtered: The number of sequences remaining after quality filtering and other processing steps. denoisedF: The number of sequences remaining after denoising of the forward primer. denoisedR: The number of sequences remaining after denoising of the reverse primer. merged: The number of sequences remaining after merging of forward and reverse reads. nonchim: The number of non-chimeric sequences, obtained after removing chimeric sequences. exclude: The number of sequences remaining after excluding sequences classified as Eukarya, Chloroplasts, or Mitochondria, as well as those of unknown Kingdom origin.Appendix S3 Behaviors and their definitions in giant panda behavior analysis

| Behavior | Type | Definition |
| --- | --- | --- |
| Feeding | D | The act of consuming various types of food, including drinking water, bamboo, bread, carrots, apples, etc. |
| Resting | D | The animal is in a stationary state with eyes closed or open |
| Moving | D | Short-distance back and forth movement in different directions or continuous non-mechanical movement |
| Investigating | F | Intently staring and slowly approaching a target object, with a distance of the nose greater than 10 cm from the target |
| Sniffing | F | The behavior occurs with the individual's nostrils dilated, with the distance between the nose and the target object less than 10 cm |
| Rubbing anogenital | F | Rubbing the anogenital region against walls, protrusions, or the ground in an arc or straight line, leaving scent marks |
| Playing | D | Purposeless play, engaging with objects or climbing |
| Grooming | F | Comfort behavior primarily involving licking, grooming (paws), scratching, and relieving itchiness |
| Stereotypic behavior | F | Repetitive actions without apparent function or purpose, including pacing, head shaking, tongue flicking, climbing on enclosure bars, etc. |
| Excretion faeces | F | Defecating |
| Gazing opposite sex | D | In a stationary state, facing the opposite sex in the neighboring enclosure, observing and following their movements |
| Urine marking | F | Marking with one leg or while standing inverted |

Note: D represents the duration of the behavior, and F represents the frequency of occurrence.


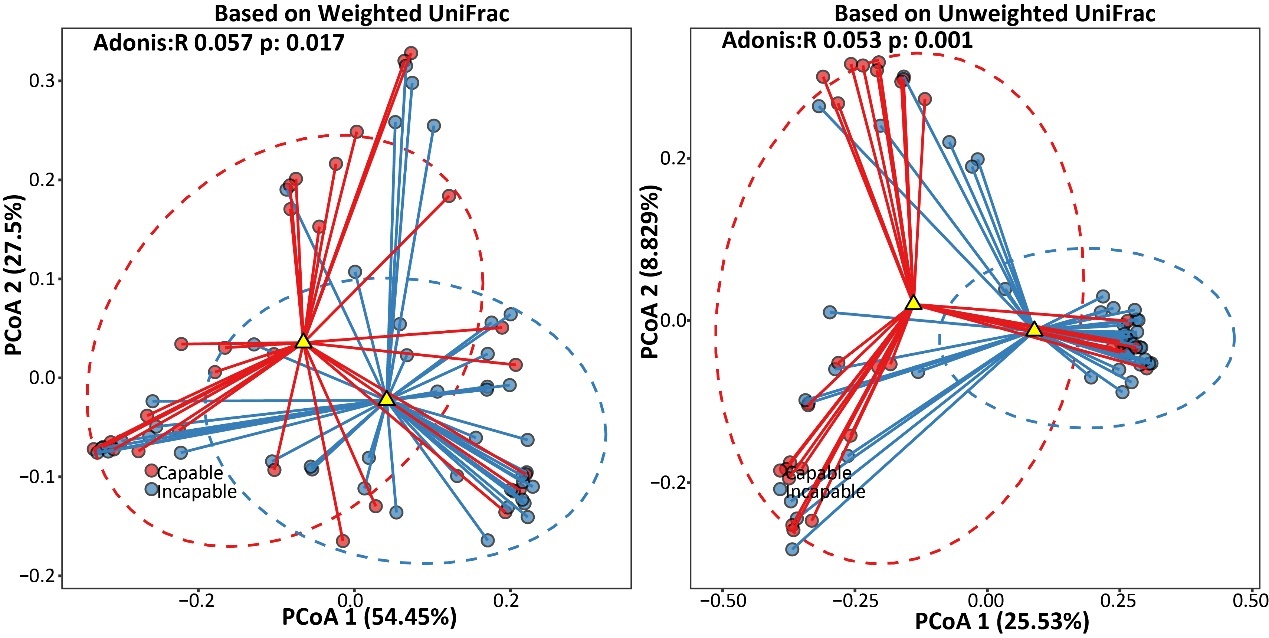


Appendix S4. Beta diversity PCoA plot based on Weighted UniFrac and Unweighted UniFrac distances, and inter-group Adonis dissimilarity index. P < 0.05 indicates significant differences.


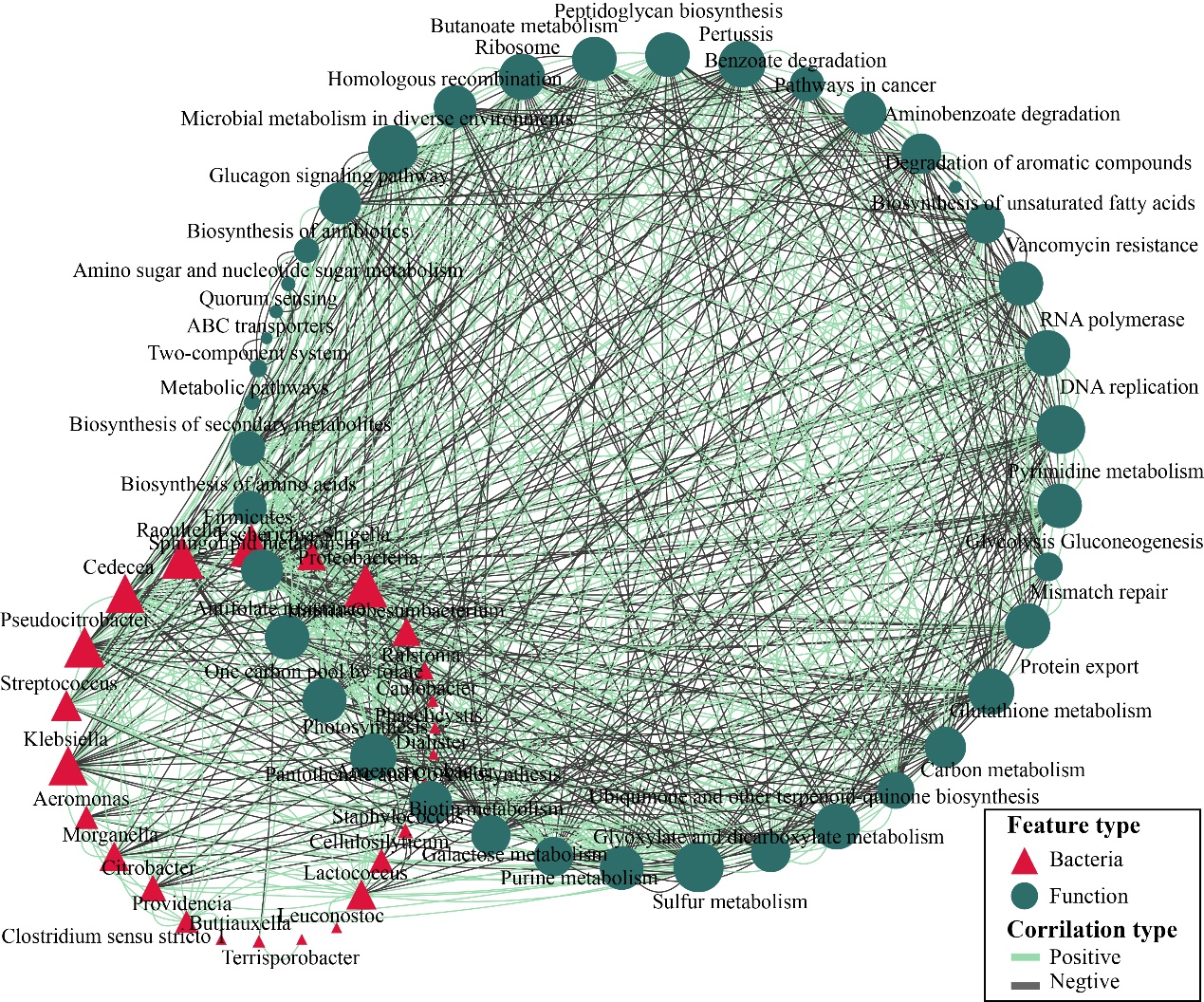


Appendix S5. Correlation between the gut microbiota and gene predicted functions in captive adult male giant pandas. The correlations were calculated using Spearman's rank correlation coefficient, and significance was determined with a p-adjust value of less than 0.05. Node size represents the relative abundance, with larger nodes indicating higher abundance.
